# A Novel Schiff Base Probe Based on Fluorescein for Fluorometric and Colorimetric Dual-Mode Rapid Detection of Cu^2+^

**DOI:** 10.3390/molecules30183824

**Published:** 2025-09-20

**Authors:** Zhi Yang, Chaojie Lei, Qian Wang, Yonghui He, Senlin Tian

**Affiliations:** 1 Faculty of Environmental Science and Engineering, Kunming University of Science and Technology, Kunming 650500, China; ymu777@163.com; 2 National and Local Joint Engineering Research Center for Green Preparation Technology of Biobased Materials, Yunnan Minzu University, Kunming 650500, China; xatutougao@163.com (C.L.); heyonghui@ymu.edu.cn (Y.H.)

**Keywords:** Cu^2+^ detection, fluorometric sensor, colorimetric sensor, fluorescein

## Abstract

Copper is an important environmental pollutant that poses a significant threat to human health and environmental safety. Therefore, the development of methods for detecting Cu^2+^ is of great significance. A novel fluorometric/colorimetric dual-mode sensor for detecting Cu^2+^ was synthesized by Schiff base reaction using fluorescein hydrazide and 8-hydroxyjulonidine-9-carboxaldehyde as raw materials. Cu^2+^ could form a complex with the probe in a stoichiometric ratio of 1:1. Within 1 min, the fluorescence of the probe rapidly quenched at 540 nm, and the absorbance reached a stable state at 452 nm. The color of the solution changed from light yellow to yellow, achieving real-time and visual detection of Cu^2+^. This probe exhibited exceptional selectivity for Cu^2+^. Within the range of 0–12 μM, the fluorescence intensity of the probe demonstrated a strong linear correlation with the concentration of Cu^2+^ (R^2^ = 0.994), with a detection limit of 0.22 µM. In the ultraviolet colorimetric method, when the Cu^2+^ concentration reached 14 μM, the absorbance stabilized (R^2^ = 0.996), and the detection limit for Cu^2+^ was determined to be 0.38 µM. Furthermore, this probe enabled reversible detection of Cu^2+^, and its performance in real water sample analysis and cellular bioimaging was proven to be highly satisfactory.

## 1. Introduction

Copper is the third most abundant essential trace element in the human body and plays a crucial role in numerous biological processes. Maintaining optimal health necessitates the presence of an appropriate concentration of Cu^2+^ ions [1,2,3]. Extensive research has demonstrated that both excess and deficiency of Cu^2+^ ions can exert toxic side effects on the human body, potentially leading to diseases such as Parkinson’s disease and Menkes syndrome [4,5,6,7,8,9,10]. Notably, the widespread use of copper in industry, agriculture, and daily life has led to its emergence as a significant environmental pollutant, posing serious threats to human health and environmental safety [11,12]. Therefore, accurate detection of Cu^2+^ is of great practical significance.

To achieve accurate detection of Cu^2+^, researchers have continuously developed and refined various detection methods. Currently, the primary techniques for detecting metal ions include inductively coupled plasma mass spectrometry (ICP-MS) [13,14,15], atomic absorption spectroscopy (AAS) [16,17,18], and electrochemical methods [19,20]. However, most of these approaches require expensive instrumentation, complex sample preparation procedures, and intricate detection processes, which limit their widespread application. In contrast, fluorescence detection [21,22,23] and UV-Vis colorimetric methods [24,25,26,27] have garnered increasing attention in environmental monitoring due to their high sensitivity, excellent selectivity, low cost, and operational simplicity [28,29,30]. In particular, small organic molecules with simple structures and low costs have attracted significant interest as chemical sensors for heavy metal ions, demonstrating both high selectivity and sensitivity [31].

**Scheme 1:**
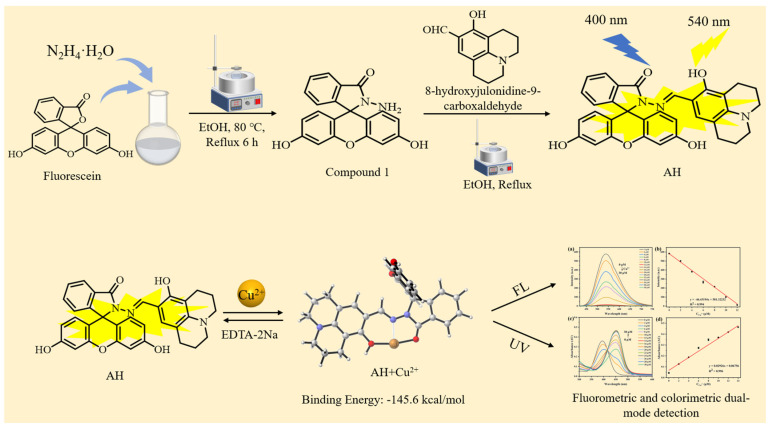
Schematic illustration of the synthesis process of probe AH and its application in Cu^2+^ detection.

Among various molecular compounds, Schiff base substances exhibit remarkable advantages in the detection of heavy metal ions. This is attributed to their straightforward synthesis process, the presence of lone electron pairs on the nitrogen atom within the imine bond in their structure, and their robust coordination ability with metal ions. Consequently, they have become a focal point in the research concerning the design and synthesis of metal ion probes. Furthermore, in the development of small molecule probes, the choice of fluorophores plays a critical role [31,32,33]. Notably, fluorescein and its derivatives are frequently employed as key materials for fabricating metal ion detection probes, owing to their superior photochemical properties, including high fluorescence quantum yield, extended absorption and emission wavelengths, excellent photostability, favorable biocompatibility, and minimal toxicity [34,35,36]. In this study, we synthesized a novel dual-mode colorimetric and fluorescent probe using fluorescein hydrazide and 8-hydroxyjulonidine-9-carboxaldehyde as raw materials. The synthetic route of probe AH and its application in Cu^2+^ detection are schematically summarized in Scheme 1. This probe not only enables visual recognition of Cu^2+^ through color changes but also allows for direct detection of Cu^2+^ through significant fluorescence changes. Furthermore, spectral performance tests indicate that this probe can rapidly identify Cu^2+^ within 1 min, demonstrating superior detection speed compared to existing reports [23,24,25,26,27]. Additionally, the probe exhibits satisfactory detection performance in real water samples and cellular bioimaging, making it a promising dual-mode sensor for the rapid detection of Cu^2+^.

## 2. Results

### 2.1. Feasibility Study of Probe AH

**Figure 1:**
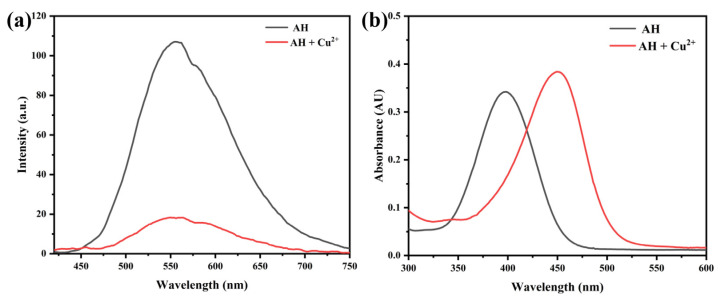
Fluorescence spectra (**a**) and UV-vis absorption spectra (**b**) of probe AH before and after addition of Cu^2+^.

To verify the dual-mode detection capability of probe AH for Cu^2+^, preliminary experiments were conducted to investigate the UV-Vis and fluorescence spectral changes in a solution containing 10 µM probe AH, 12 µM Cu^2+^, and an ethanol/HEPES buffer (3:7, *v*/*v*, 20 mM HEPES, pH 7.0) before and after the addition of Cu^2+^. As illustrated in Figure 1, the probe AH blank solution exhibited a UV absorption peak at 400 nm. Upon the addition of Cu^2+^, this peak disappeared, and a new absorption peak emerged at 452 nm. When excited at 400 nm, the fluorescence emission spectrum revealed that the probe AH alone displayed strong fluorescence, whereas the fluorescence intensity at 562 nm decreased significantly upon Cu^2+^ addition, approaching nearly zero. These results indicate that probe AH reacts with Cu^2+^, resulting in distinct changes in both fluorescence and UV-Vis absorption spectra. This confirms that probe AH can be effectively utilized for dual-mode fluorescent and colorimetric detection of Cu^2+^.

### 2.2. Optimization of Detection Conditions

In order to achieve the best detection effect of Cu^2+^ by probe AH, important factors such as solvent type, solvent ratio, pH, and response time were optimized.

#### 2.2.1. Effect of Solvent Type on the Luminescence Properties of Probe AH

**Figure 2:**
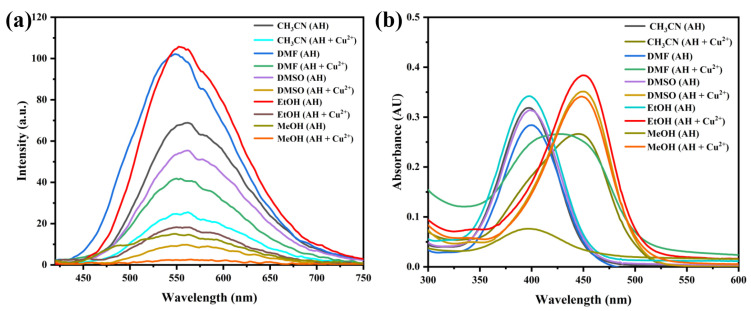
Fluorescence spectra (**a**) and UV-vis absorption spectra (**b**) of probe AH before and after Cu^2+^ addition in different solvent systems.

The luminescence characteristics of the probe exhibit significant variations across different solvents. An appropriate organic solvent can effectively prevent aggregation, suppress undesirable side reactions, and enhance the probe’s detection performance towards the target analyte. To identify a suitable organic solvent and improve the detection efficiency of Cu^2+^ by the probe, fluorescence and UV absorption spectra were recorded before and after Cu^2+^ (12 µM) addition in five solvents (acetonitrile, N, N-dimethylformamide, dimethyl sulfoxide, ethanol, and methanol) that are miscible with water and do not chemically react with the probe (10 µM). As shown in Figure 2, the probe displayed fluorescence and UV responses to Cu^2+^ in various solvent/HEPES (3:7, *v*/*v*, 20 mM HEPES) systems. Among them, the ethanol-based system exhibited the most pronounced changes in fluorescence intensity and absorbance, resulting in the most significant detection performance. Therefore, ethanol was selected as the optimal solvent for subsequent experiments.

#### 2.2.2. Effect of Solvent Ratio on the Luminescence Properties of Probe AH

**Figure 3:**
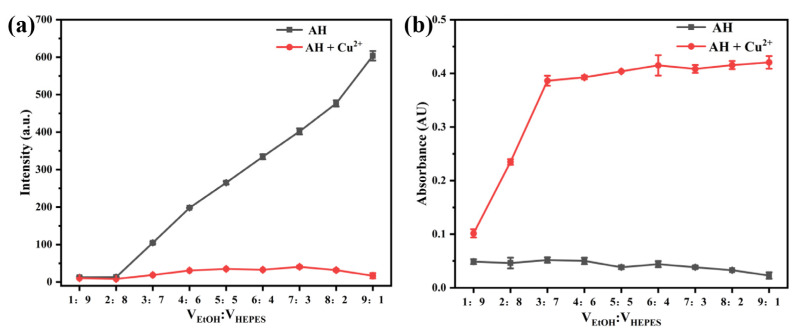
Fluorescence intensity (**a**) and absorbance (**b**) of probe AH before and after Cu^2+^ addition in EtOH/HEPES systems with different volume ratios.

Following the confirmation of ethanol as the optimal solvent, we further investigated the changes in fluorescence intensity and UV absorbance of the probe (10 µM) before and after Cu^2+^ (12 µM) addition under varying solvent ratios. As illustrated in Figure 3, in the fluorescence spectrum, the fluorescence intensity of the probe blank solution gradually increased with the increasing volume ratio of ethanol to HEPES. Upon the addition of Cu^2+^, the fluorescence intensity decreased significantly and stabilized. In UV spectroscopy, the UV absorbance of the probe blank solution remained relatively constant across different solvent ratios. However, after Cu^2+^ addition, the absorbance stabilized when the ethanol/HEPES ratio reached 3:7. To achieve optimal results in both fluorescence and UV detection, subsequent experiments were conducted at a volume ratio of 9:1 between ethanol and HEPES.

#### 2.2.3. The Effect of pH on the Luminescence Performance of Probe AH

**Figure 4:**
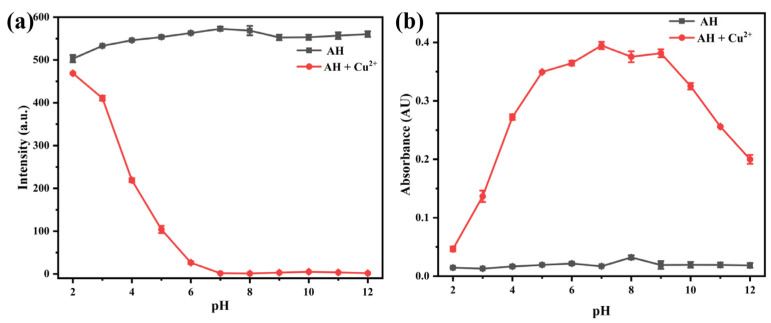
Fluorescence intensity (**a**) and absorbance (**b**) of probe AH before and after Cu^2+^ addition at different pH values.

In order to investigate the influence of solution pH on the luminescence performance of the probe, the changes in fluorescence intensity and absorbance of the probe during the detection process in the EtOH/HEPES (9:1, *v*/*v*, 20 mM HEPES) system with different pH values after adding Cu^2+^ (12 µM) were examined after the mixture of ethanol and HEPES buffer solution. As shown in Figure 4a, the fluorescence intensity of the probe’s blank solution remains stable within the pH range of 2–12, exhibiting a pronounced fluorescence response. Upon the introduction of Cu^2+^, the fluorescence intensity progressively diminishes with increasing pH. Within the pH range of 7–12, the fluorescence intensity approaches zero and stabilizes, demonstrating an excellent quenching effect.

Simultaneously, as depicted in Figure 4b, the probe blank solution exhibited stable behavior within the pH range of 2–12. After Cu^2+^ addition, the absorbance at 452 nm gradually increased with rising pH and stabilized within the pH range of 5–9. When pH ≥ 9, the absorbance gradually decreased, likely due to the formation of Cu(OH)_2_ precipitate under alkaline conditions.

These experimental results indicate that the probe possesses good pH adaptability, making it a promising candidate for detecting Cu^2+^ in natural environments and biological systems, which are typically neutral or slightly alkaline. Therefore, subsequent experiments were conducted under pH 7.0 conditions to simulate these environments.

#### 2.2.4. Response Time of Probe AH to Cu^2+^

**Figure 5:**
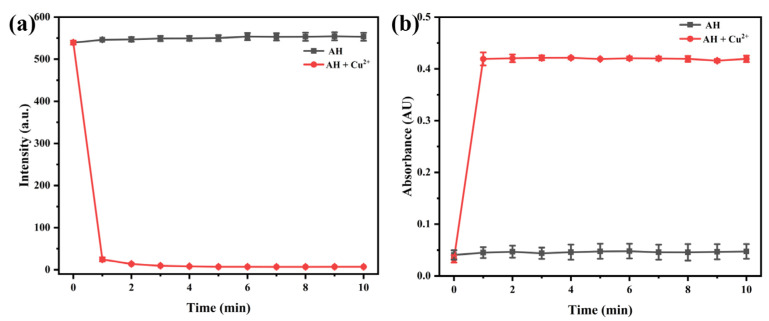
Changes in fluorescence intensity (**a**) and absorbance (**b**) of probe AH over time before and after the addition of Cu^2+^.

Response time is a critical parameter for evaluating the performance of a probe. To assess the real-time detection capability for Cu^2+^ (12 µM), we monitored the time-dependent changes in fluorescence intensity (540 nm) and absorbance (452 nm) before and after Cu^2+^ addition. As shown in Figure 5, prior to Cu^2+^ addition, the fluorescence intensity of the probe blank solution at 540 nm and the absorbance at 452 nm remained stable. Upon Cu^2+^ addition, the probe rapidly reacted with Cu^2+^, causing the fluorescence intensity to decrease sharply and stabilize within 1 min, while the UV absorbance increased rapidly and gradually stabilized within the same timeframe. These results demonstrate that the probe is capable of real-time detection of Cu^2+^.

### 2.3. Concentration Titration Experiments of Probe AH with Cu^2+^

In order to assess the sensitivity of the probe AH toward Cu^2+^, the fluorescence spectra and ultraviolet absorption spectra of the probe were analyzed after adding varying concentrations of Cu^2+^ under optimal conditions. Specifically, multiple 5 mL centrifuge tubes were prepared. To each tube, 40 μL of a 1 mM probe stock solution was added. Subsequently, a 10 mM Cu^2+^ stock solution was added to each tube according to a defined concentration gradient. The solutions were then diluted to 4 mL using an EtOH/HEPES buffer (9:1, *v*/*v*, 20 mM HEPES, pH 7.0) to achieve final Cu^2+^ concentrations ranging from 0 to 30 μM. Changes in the fluorescence spectra of these solutions were subsequently recorded.

**Figure 6:**
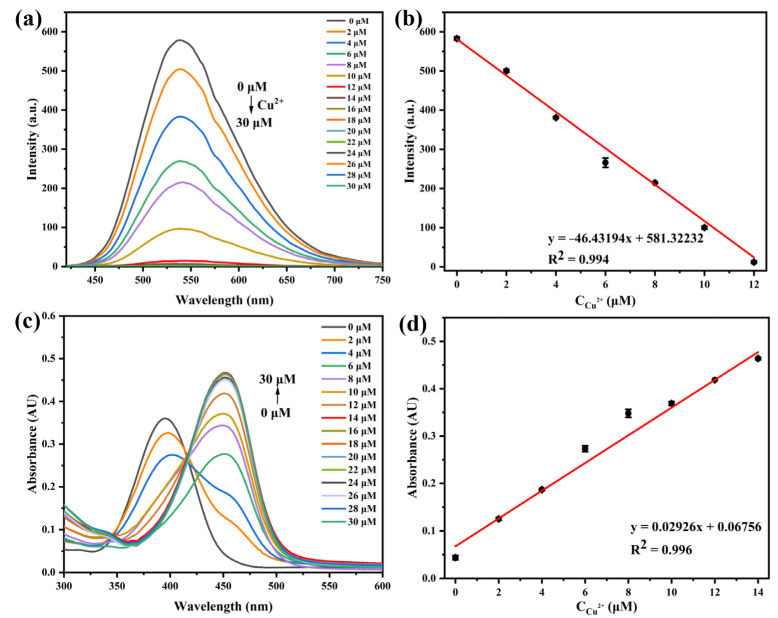
(**a**) Fluorescence spectra of probe AH at different concentrations of Cu^2+^ (0–30 μM); (**b**) The linear relationship between the fluorescence intensity of probe AH and the concentration of Cu^2+^ (0–12 μM); (**c**) UV absorption spectra of probe AH at different concentrations of Cu^2+^ (0–30 μM); (**d**) Linear relationship between absorbance of probe AH and Cu^2+^ concentration (0–14 μM).

As shown in Figure 6a,b, the maximum emission wavelength of the fluorescence spectrum was 540 nm. Upon the addition of Cu^2+^, the fluorescence intensity at this wavelength decreased progressively with increasing Cu^2+^ concentration. When the Cu^2+^ concentration reached 12 µM, the fluorescence intensity plateaued. Moreover, within the concentration range of 0–12 µM, the fluorescence response of the probe to Cu^2+^ exhibited a strong linear relationship (R^2^ = 0.994), with a linear equation of y = −46.43194x + 581.32232. Based on the detection limit calculation formula LOD = 3σ/k, the detection limit for Cu^2+^ was determined to be 0.22 µM.

The UV-Vis absorption spectra of the system were recorded using the same method, as shown in Figure 6c,d. In the UV spectrum, the absorbance at 400 nm decreased gradually with increasing Cu^2+^ concentration, while a new absorption peak emerged at 452 nm, with its absorbance increasing with Cu^2+^ concentration. When the Cu^2+^ concentration reached 14 µM, the absorbance stabilized, and a distinct isosbestic point appeared at 420 nm. Within the concentration range of 0–14 µM, the probe exhibited a strong linear relationship (R^2^ = 0.996) for the UV response to Cu^2+^, with a linear equation of y = 0.02926x + 0.06756. Based on the detection limit calculation formula LOD = 3σ/k, the detection limit for Cu^2+^ was determined to be 0.38 µM.

These results demonstrate that probe AH exhibits high sensitivity towards Cu^2+^, with low detection limits, making it a promising candidate for the detection of Cu^2+^ in various applications.

### 2.4. Selectivity Study of Probe AH for Cu^2+^ Detection

Selectivity is a crucial parameter for assessing the performance of a probe. To determine whether the probe exhibits specific recognition of Cu^2+^, a selectivity experiment was conducted under optimal detection conditions. Initially, multiple centrifuge tubes were prepared, and 40 µL of 1 mM probe stock solution was added to each tube. Subsequently, 4.8 µL of 10 mM solutions of various metal ions (Ag^+^, Al^3+^, Ba^2+^, Ca^2+^, Cd^2+^, Co^2+^, Cr^3+^, Cu^2+^, Hg^2+^, Fe^2+^, Fe^3+^, K^+^, Mg^2+^, Mn^2+^, Na^+^, Ni^2+^, Pb^2+^, and Zn^2+^) were individually added to each centrifuge tube. The solutions were then diluted to a final volume of 4 mL using a mixture of ethanol and HEPES buffer solution (9:1, *v*/*v*, 20 mM HEPES, pH 7.0). Additionally, a 10 µM probe solution was prepared as a blank control. After stabilization, the fluorescence and UV-Vis absorption spectra of the solutions were measured.

**Figure 7:**
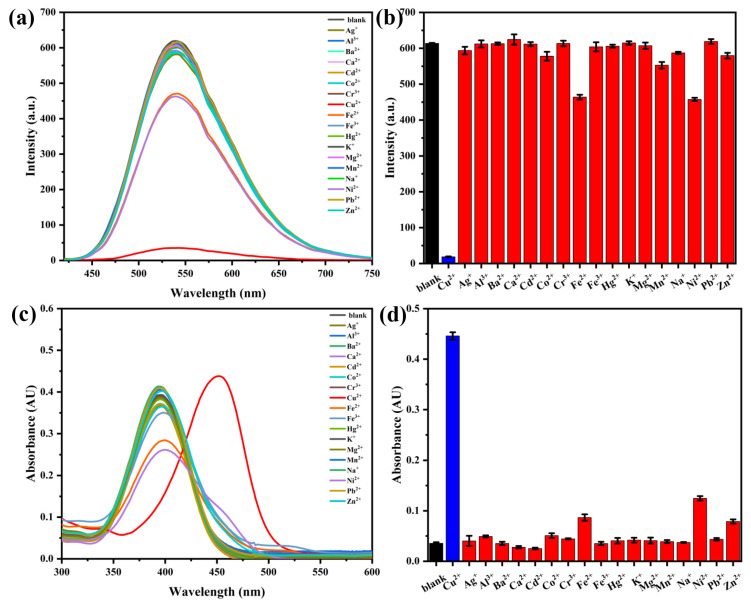
Fluorescence spectra (**a**) and ultraviolet absorption spectra (**c**) of probe AH mixed with different metal ions; fluorescence intensity at 540 nm (**b**) and absorbance at 452 nm (**d**) of probe AH mixed with different metal ions.

As shown in Figure 7a,b, the probe exhibited a strong emission peak at 540 nm in the absence of metal ions. Upon the addition of Cu^2+^, the fluorescence intensity at 540 nm sharply decreased. The UV-Vis spectra (Figure 7c) revealed a characteristic absorption peak at 400 nm before the addition of Cu^2+^, which disappeared upon Cu^2+^ addition, concomitant with the emergence of a new peak at 452 nm. In contrast, the addition of other metal ions did not induce significant changes in either fluorescence or UV-Vis absorption spectra. Collectively, these results demonstrate that the probe possesses high selectivity for Cu^2+^.

### 2.5. Interference Resistance Study of Probe AH for Cu^2+^ Detection

**Figure 8:**
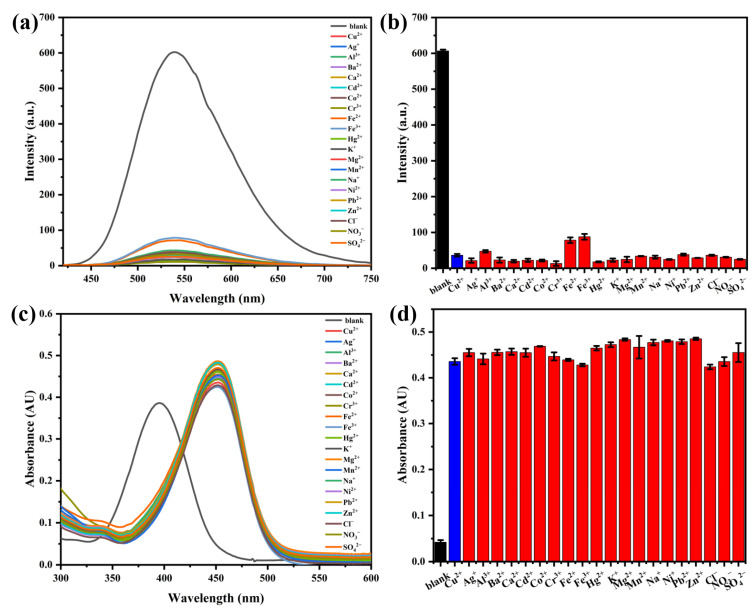
Fluorescence spectra (**a**) and ultraviolet absorption spectra (**c**) of probe AH mixed with different metal ions in the presence of Cu^2+^; fluorescence intensity at 540 nm (**b**) and absorbance at 452 nm (**d**) of probe AH mixed with different metal ions in the presence of Cu^2+^.

Achieving specific recognition of the target object in a complex environment is an important parameter for evaluating probes. Therefore, this study investigates the anti-interference capability of the probe AH. First, multiple 5 mL centrifuge tubes were prepared. Subsequently, 40 μL of probe stock solution (1 mM) was added to each tube. Then, 4.8 μL of Cu^2+^ solution (10 mM) was introduced into the probe solution. Additionally, reserve solutions of various ions (1 mM, including Ag^+^, Al^3+^, Ba^2+^, Ca^2+^, Cd^2+^, Co^2+^, Cr^3+^, Hg^2+^, Fe^2+^, Fe^3+^, K^+^, Mg^2+^, Mn^2+^, Na^+^, Ni^2+^, Pb^2+^, Zn^2+^, Cl^−^, NO_3_^−^, and SO_4_^2−^) were separately added to the respective solutions. The final volume was adjusted to 4 mL by adding a mixture of EtOH and HEPES buffer (9:1, *v*/*v*, 20 mM HEPES, pH = 7.0). A blank solution of probe-Cu^2+^ was also prepared under the same conditions. After allowing the solutions to stabilize, the anti-interference capability of the probe towards Cu^2+^ was assessed by monitoring fluorescence and UV-Vis spectra. As depicted in Figure 8, the probe maintained its response to Cu^2+^ in the presence of other metal ions, with minimal changes in fluorescence intensity and absorbance. These results demonstrate that the probe exhibits high selectivity and robust anti-interference ability for Cu^2+^, making it a promising candidate for the detection of Cu^2+^ in complex environments.

### 2.6. Reversibility Study of Probe AH

**Figure 9:**
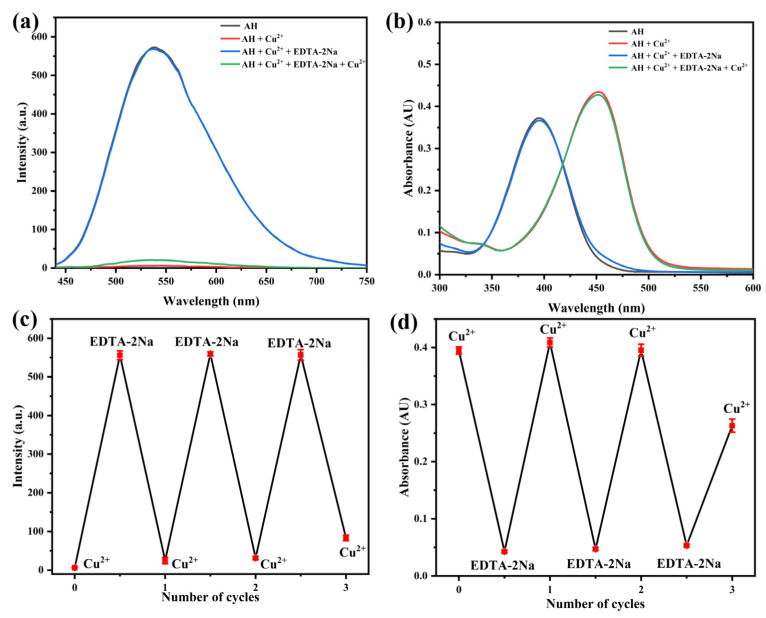
(**a**) Investigation of the reversibility of EDTA-2Na in the fluorescence spectrum of probe AH; (**b**) Exploration of the reversibility of EDTA-2Na in the UV absorption spectra of probe AH; (**c**) The reversible cycle number of Cu^2+^ detected by probe AH fluorescence; (**d**) Probe AH UV detection of the reversible cycle number of Cu^2+^.

Reversibility is one of the important properties of probes. To verify the reversibility of the reaction between the probe and Cu^2+^, titration experiments were conducted using chelating agents (disodium ethylenediaminetetraacetic acid, EDTA-2Na) in an EtOH/HEPES mixed system (9:1, *v*/*v*, 20 mM HEPES, pH 7.0). As shown in Figure 9a,b, upon the addition of the chelating agent (12 μM) to the probe-Cu^2+^ solution, the fluorescence intensity at 540 nm rapidly increased, while the absorbance at 452 nm rapidly decreased. Subsequently, upon reintroducing Cu^2+^ (12 μM), the fluorescence intensity of the mixed solution rapidly decreased, and the absorbance rapidly increased. These observations indicate that the recognition process of Cu^2+^ by the probe is reversible.

Moreover, as depicted in Figure 9c,d, the probe exhibited the capacity for three cycles of reversible binding and release during the sequential addition of Cu^2+^ and EDTA-2Na. This finding underscores the probe’s potential for repeated use in Cu^2+^ detection, a highly desirable feature for practical applications.

### 2.7. Study on the Recognition Mechanism of Cu^2+^ by Probe AH

**Figure 10:**
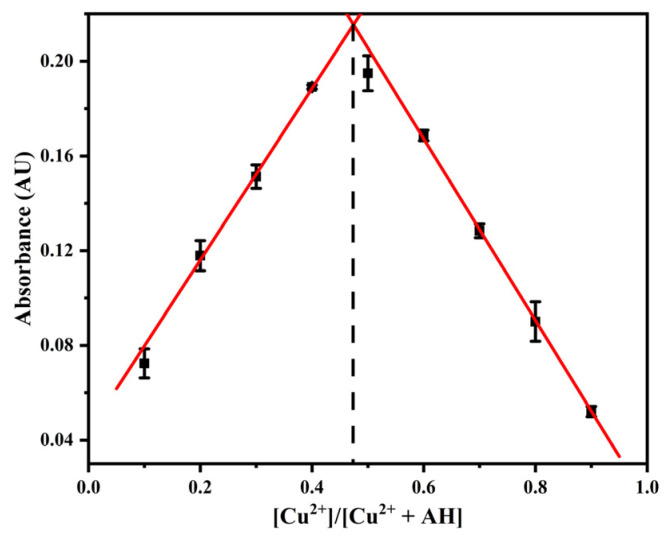
Job’s plot curve between probe AH and Cu^2+^.

To determine the stoichiometric ratio of probe AH binding to Cu^2+^, Job’s plot curve experiments were conducted. The total concentration of probe AH and Cu^2+^ was maintained at 10 µM in an EtOH/HEPES buffer solution (9:1, *v*/*v*, 20 mM HEPES, pH 7.0), while the molar fraction of Cu^2+^ was systematically varied. The absorbance of the probe at 452 nm was measured at different Cu^2+^ molar fractions, and a plot was constructed with the molar fraction of Cu^2+^ on the *x*-axis and the corresponding absorbance on the *y*-axis. As shown in Figure 10, the intersection point of the curve is located near a molar fraction of 0.5, indicating that the probe binds to Cu^2+^ in a 1:1 stoichiometric ratio.

**Figure 11:**
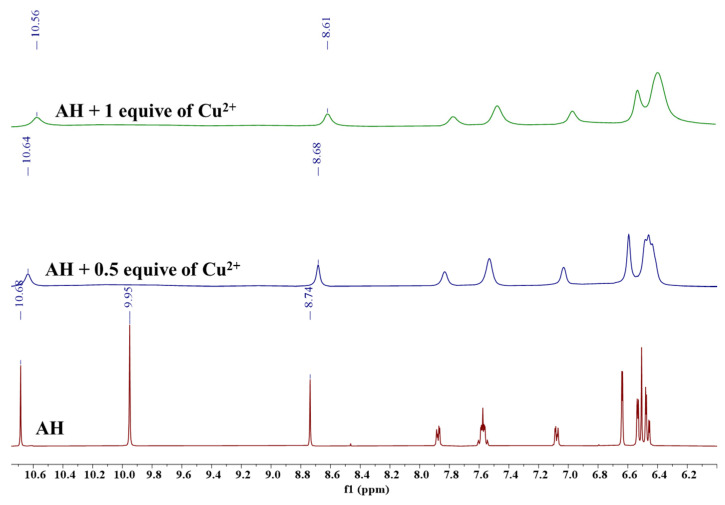
^1^H NMR spectra of fluorescent probes AH and AH-Cu^2+^.

To further elucidate the complexation mechanism between probe AH and Cu^2+^, nuclear magnetic resonance (NMR) titration experiments were conducted in deuterated dimethyl sulfoxide (DMSO-d_6_), and the results are presented in Figure 11. Prior to complexation with Cu^2+^, the proton signals of the hydroxyl group and the carbon-nitrogen double bond in probe AH were observed at 10.68 ppm. Specifically, the hydroxyl proton on 8-hydroxyjulonidine-9-carboxaldehyde resonated at 9.95 ppm, while the proton on the carbon-nitrogen double bond appeared at 8.74 ppm. Upon complexation with Cu^2+^, the chemical shifts of the protons on the carbon-nitrogen double bond, initially at 10.68 ppm and 8.74 ppm, shifted downfield to 10.01 ppm and 8.60 ppm, respectively. These observations suggest significant structural changes in the aromatic ring of probe AH upon Cu^2+^ binding.

**Figure 12:**
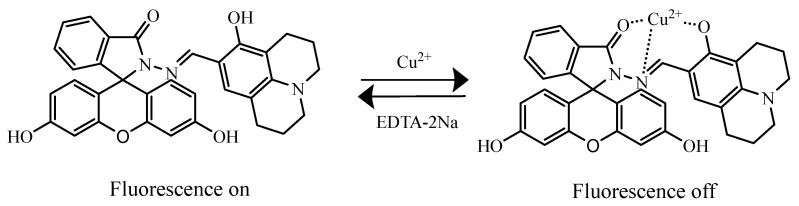
The recognition mechanism diagram of probe AH for Cu^2+^.

The density functional theory (DFT) calculations [37,38,39,40,41,42,43,44,45] results indicate that copper ions exhibit a relatively high binding energy (−145.6 kcal/mol) when coordinating with oxygen atoms from both hydroxyl (OH) and carbonyl (C=O) groups in a 1:1 stoichiometric ratio. Detailed computational data can be found in the Supplementary Materials. To further clarify the binding mechanism between probe AH and Cu^2+^, we integrated experimental results with DFT calculations to propose a plausible binding mode between AH and Cu^2+^, as illustrated in Figure 12.

**Figure 13:**
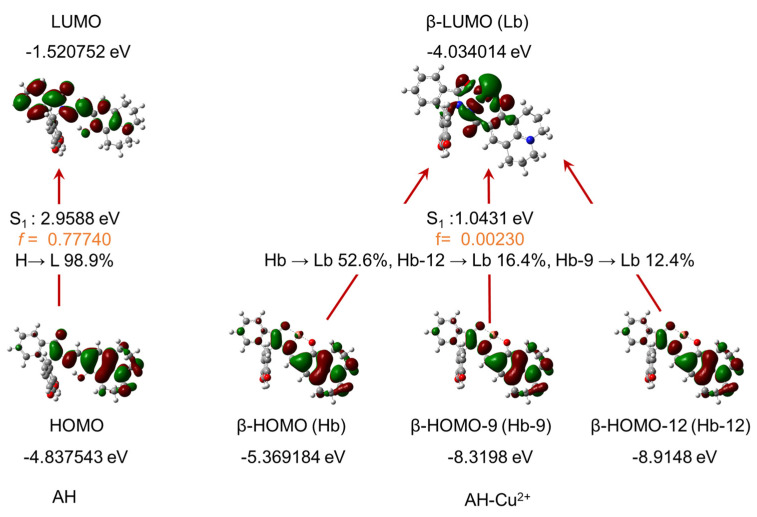
TD-DFT calculations reveal the mechanisms of probe AH fluorescence emission and quenching.

Generally, the open form of fluorescein exhibits strong fluorescence emission, whereas the closed form demonstrates extremely weak emission. However, the anomalous fluorescence behavior observed in this system warranted further investigation. To elucidate this phenomenon, we performed TD-DFT calculations; the results are presented in Figure 13. For probe AH, the oscillator strength (f) of its first excited state (S_1_) is 0.7774, confirming it as a bright (fluorescent) state. Upon binding Cu^2+^ ions, the S_1_ oscillator strength significantly decreases to 0.0023, indicating a transition to a dark (non-fluorescent) state. This computational result aligns perfectly with the experimental observation of Cu^2+^-induced fluorescence quenching in AH. Analysis of AH’s electronic structure reveals that its S_1_ state arises predominantly (98.9%) from the HOMO→LUMO transition. This excitation process involves electron transfer primarily between the julolidine moiety and the spirobenzene ring of the fluorescein core, characteristic of an intramolecular charge transfer (ICT) process. Crucially, this electron transfer pathway does not involve the characteristic opening of the fluorescein spirocycle, diverging from the classic open-closed (spirocyclization) mechanism. Electronic structure analysis of the AH-Cu complex clarifies its quenching mechanism. As an open-shell system, the S_1_ state of AH-Cu comprises contributions from the β-LUMO and several occupied orbitals (β-HOMO, β-HOMO-9, β-HOMO-12). The resulting electron transfer is localized onto the Cu center. This indicates that the electron-deficient Cu^2+^ ion acts as an efficient electron acceptor, facilitating fluorescence quenching via a photoinduced electron transfer (PET) process.

Currently, the majority of fluorescent probes developed for Cu^2+^ sensing demonstrate a fluorescence “turn-on” response, as opposed to a quenching-based mechanism [18,46,47]. Compared with “turn-on” fluorescent probes, the fluorescence sensing strategy based on the quenching mechanism offers several distinct advantages. First, it provides high sensitivity and low background interference: fluorescence quenching probes exhibit strong initial fluorescence signals in the absence of the target, thereby generating a high signal-to-noise ratio (S/N), which is particularly suitable for the detection of low-concentration analytes. Moreover, the strategy demonstrates excellent photostability: since the detection relies on the reduction rather than the enhancement of fluorescence signals, continuous excitation of the fluorophore is not required, thereby minimizing photobleaching and making it well-suited for long-term dynamic monitoring.

### 2.8. Visual Detection of Cu^2+^ by Probe AH

**Figure 14:**
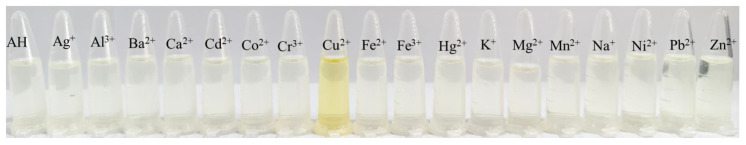
Color changes in probe AH after adding different metal ions under sunlight.

**Figure 15:**
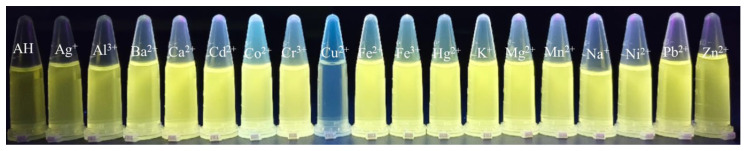
Color changes in probe AH after adding different metal ions under UV lamp (365 nm).

The ability to visually observe color changes in a probe upon the addition of a target analyte is a crucial indicator of the performance of fluorescent probes. To evaluate this aspect, a visualization study was conducted using the fluorescent probe AH. As shown in Figure 14 and Figure 15, the color of probe AH solutions containing various metal ions was examined under both daylight and ultraviolet (UV) irradiation. Only the probe solution with added Cu^2+^ exhibited a noticeable color change. Under daylight, the solution’s color shifted from light yellow to a deeper yellow. Under UV irradiation, the original yellow fluorescence of the probe was quenched upon the addition of Cu^2+^. In contrast, the presence of other metal ions did not induce any color changes in the probe AH solution. These results demonstrate that the fluorescent probe AH can specifically recognize Cu^2+^ through a visually observable response, highlighting its potential for naked-eye detection of Cu^2+^.

### 2.9. Analysis of Real Samples and Cellular Bioimaging

To evaluate the practical applicability of this method in real-world sample analysis, the probe was employed to determine the Cu^2+^ content in water samples collected from various environmental sources. As detailed in Table 1 and Table 2, the relative standard deviation (RSD) of Cu^2+^ detection in different environmental water samples using fluorescence was less than 4.00%, with average recovery rates ranging from 93.00% to 107.00%. Similarly, when using UV detection, the RSD of Cu^2+^ in these samples was less than 4.98%, and the average recovery rates were between 99.25% and 105.88%. Both methods yielded satisfactory results, demonstrating that the probe exhibits excellent sensitivity and accuracy in the analysis of real water samples. These findings highlight the practical application value of the probe for Cu^2+^ detection in complex environmental matrices.

To investigate the potential biological applications of probe AH, its cytotoxicity was assessed using the MTT assay. As shown in Figure S9, even at a concentration of 50 μM, the viability of HepG2 cells remained above 85%, indicating low cytotoxicity. Laser scanning confocal microscopy (LSCM) results demonstrated that AH was capable of entering HepG2 cells without inducing noticeable morphological changes. More importantly, under fluorescence detection conditions, HepG2 cells exhibited intense yellow fluorescence (Figure S10a–c), suggesting efficient cellular uptake of AH. Upon the addition of copper ions, the fluorescence signal in HepG2 cells was markedly reduced (Figure S10d–f). Collectively, these findings indicate that AH not only exhibits favorable biocompatibility and minimal cytotoxicity but also demonstrates a robust response to intracellular copper ions.

### 2.10. Comparison with Other Probes

During the experiment, we systematically analyzed and summarized the ε, λmax, λex, λem, Stokes shift, and Φ values for both probe AH and the AH-Cu^2+^ solution to more effectively illustrate their spectral characteristics. The detailed data are presented in Table S1 of Supplementary Materials. Furthermore, we compared this probe with other probes for detecting Cu^2+^, and the results are shown in Table 3. The comparative results demonstrate that probe AH exhibits superior performance in the dual-mode Fluorometric and colorimetric detection of Cu^2+^, with a rapid response time and an exceptionally low detection limit.

## 3. Materials and Methods

### 3.1. Reagents and Instruments

All reagents used in the experiments were of analytical grade and were used without further purification. Fluorescein, hydroxyethyl piperazine ethanethiol sulfonic acid (HEPES), and the ionic salts were purchased from McLean Biochemical Technology Co., Ltd., Shanghai, China. 8-hydroxyjulonidine-9-carboxaldehyde was obtained from Titan Technology Co., Ltd., Shanghai, China. Hydrazine hydrate, glacial acetic acid, anhydrous ethanol, dimethyl sulfoxide (DMSO), acetonitrile, tetrahydrofuran, and *N*,*N*-dimethylformamide were all purchased from Cologne Chemical Co., Ltd., Chengdu, China.

Experimental equipment: High-resolution mass spectra were obtained using a BRUKER ESI-Q-TOF mass spectrometer. Proton nuclear magnetic resonance (^1^H NMR) and carbon-13 nuclear magnetic resonance (^13^C{^1^H} NMR) spectra were recorded using a BRUKER AV III-400 NMR spectrometer, with tetramethylsilane (TMS) as the internal standard. Fourier transform infrared (FTIR) spectroscopy measurements were performed using a NICOLET iS10 spectrometer. UV-Vis and fluorescence spectra were measured using a Cary 8454 UV-Vis spectrophotometer and a Cary Eclipse fluorescence spectrophotometer, respectively.

### 3.2. Synthesis of Compound ***1***

In accordance with the literature procedure, fluorescein (1.66 g, 5 mmol) was introduced into a three-necked flask containing 50 mL of ethanol. Excess hydrazine hydrate (2.5 mL) was then slowly added to the mixture. The reaction mixture was heated to 80 °C and refluxed while stirring for 6 h to obtain a dark red transparent solution. The dark red solution was subsequently subjected to rotary evaporation until a small amount of solid was formed. The resulting mixture was then poured into an appropriate volume of deionized water and allowed to stand until precipitation occurred. The precipitated solid was filtered, washed with deionized water until the filtrate became colorless, and then washed with anhydrous ethanol to obtain the crude product. The crude product was recrystallized from anhydrous ethanol and vacuum-dried. The dried light yellow solid was identified as compound **1**, fluorescein hydrazide, with a yield of 1.21 g (70.21%). ^1^H NMR (400 MHz, DMSO-*d*_6_) δ: 9.82 (s, 2H), 7.80–7.75 (m, 1H), 7.53–7.45 (m, 2H), 7.02–6.96 (m, 1H), 6.59 (d, *J* = 2.4 Hz, 2H), 6.48–6.37 (m, 4H), 4.39 (s, 2H). ^13^C{^1^H} NMR (100 MHz, DMSO-*d*_6_) δ: 165.98, 158.65, 152.87, 152.00, 133.08, 129.79, 128.88, 128.43, 123.90, 122.84, 112.47, 110.42, 102.83, 65.09. FT-IR (KBr Pellet, cm^−1^): 3269(-OH), 1691(-C=O), 1184(C-O-C).

### 3.3. Synthesis of Probe AH

Compound **1** (0.345 g, 1 mmol) and 8-hydroxyjulonidine-9-carboxaldehyde (0.2172 g, 1 mmol) were dissolved in 20 mL of ethanol. The reaction mixture was heated to 80 °C, and a few drops of glacial acetic acid were added as a catalyst. The solution was then refluxed and monitored using thin-layer chromatography (TLC) to track the progress of the reaction. Upon completion of the reaction, the mixture was allowed to cool to room temperature, resulting in the formation of a precipitate. The precipitate was filtered and thoroughly washed with cold ethanol. The resulting yellow solid was dried completely in an oven to obtain the final product, probe AH. The yield was 0.7363 g (67.53%). ^1^H NMR (400 MHz, DMSO-*d*_6_) δ: 10.69 (s, 1H), 9.95 (s, 2H), 8.74 (s, 1H), 7.88 (dd, *J* = 6.1, 2.7 Hz, 1H), 7.57 (m, 2H), 7.11–7.04 (m, 1H), 6.64 (d, *J* = 2.4 Hz, 2H), 6.56–6.48 (m, 4H), 6.46 (d, *J* = 2.4 Hz, 1H), 3.12 (m, 4H), 2.53 (d, *J* = 6.6 Hz, 2H), 2.43 (t, *J* = 6.5 Hz, 2H), 1.78 (s, 4H). ^13^C{^1^H} NMR (100 MHz, DMSO-*d*_6_) δ: 163.21, 159.05, 156.80, 155.08, 152.52, 151.03, 146.09, 134.01, 129.46, 129.40, 128.54, 124.03, 123.40, 113.12, 112.96, 109.91, 106.40, 105.78, 103.01, 65.42, 49.69, 49.21, 26.83, 21.77, 20.90, 20.51. FT-IR (KBr Pellet, cm^−1^): 3323(-OH), 1669(-CH=N-), 1625(-C=O). HRMS (ESI): *m*/*z* calcd for C_33_H_27_N_5_O_5_ [M + Na]^+^ 568.1843, found 568.1866.

### 3.4. UV Visible and Fluorescence Spectroscopy Study

Ionic compounds, including AgNO_3_, Al(NO_3_)_3_·9H_2_O, Ba(NO_3_)_2_, Ca(NO_3_)_2_·4H_2_O, CdCl_2_·2.5H_2_O, CoCl_2_·6H_2_O, Cr(NO_3_)_3_·9H_2_O, Cu(NO_3_)_2_·H_2_O, FeCl_2_·4H_2_O, FeCl_3_·6H_2_O, HgCl_2_, KNO_3_, Mg(NO_3_)_2_·6H_2_O, MnCl_2_·4H_2_O, NaNO_3_, Ni(NO_3_)_2_·6H_2_O, Pb(NO_3_)_2_, Zn(NO_3_)_2_·6H_2_O, NaCl, and Na_2_SO_4_ were each dissolved in deionized water to prepare 10 mM stock solutions for subsequent use. Additionally, compound AH was dissolved in ethanol to prepare a 1 mM probe stock solution at room temperature for future experiments.

For spectroscopic studies, the stock solutions were appropriately diluted to obtain the desired test solutions. Optical detection was performed in an ethanol/HEPES buffer system (9:1, *v*/*v*, 20 mM HEPES, pH 7.0). The spectral testing conditions were set as follows: The excitation wavelength was 400 nm, and the slit width for both the excitation and emission wavelengths was adjusted to 5 nm.

### 3.5. Calculation of Detection Limit

The detection limit (DL) of probe AH for Cu^2+^ is determined using the formula DL = 3σ/k, where σ represents the standard deviation of the fluorescence intensity (or UV absorbance) of the blank solution, and k is the slope of the linear relationship graph between fluorescence intensity (or UV absorbance) and Cu^2+^ concentration.

### 3.6. Analysis of Water Samples

To evaluate the practical applicability of probe AH in real-world sample analysis, the Cu^2+^ content in various environmental water samples was assessed using this method. In the experiments, tap water, river water (from Laoyu River), and lake water (from Dianchi Lake) were each filtered through 0.22 µm filter membranes to remove particulate matter. Subsequently, Cu^2+^ detection was performed on these filtered water samples. To further validate the method, a known concentration of Cu^2+^ was spiked into each real water sample using the standard addition method. The concentration and recovery rate of Cu^2+^ were then calculated based on the measured responses.

## 4. Conclusions

In summary, this study designs and synthesizes a novel dual-mode colorimetric and fluorescence “turn-off” chemical sensor, AH, based on fluorescein hydrazide, and investigates its selectivity for various metal ions. The results demonstrate that sensor AH exhibits excellent dual-mode sensing capabilities for Cu^2+^, functioning both as a colorimetric sensor for “naked-eye” detection and as a fluorescence “turn-off” sensor. Upon the addition of Cu^2+^, the solution color changes from yellow to colorless. The absorbance of the probe shows a good linear relationship with Cu^2+^ concentration in the range of 0–12 µM, with a detection limit of 0.38 µM. Additionally, the probe emits strong fluorescence at 540 nm in the absence of Cu^2+^, which is quenched upon Cu^2+^ addition. The fluorescence response also exhibits a good linear relationship with Cu^2+^ concentration in the range of 0–12 µM, with a detection limit of 0.22 µM. Job’s plot analysis confirms a 1:1 coordination ratio between the probe and Cu^2+^. Furthermore, the probe demonstrates good reusability, achieving three cycles of Cu^2+^ detection. It also shows satisfactory performance in detecting Cu^2+^ in real water samples and cellular bioimaging, highlighting its potential for practical applications.

## Figures and Tables

**Table 1:** Fluorescence detection of Cu^2+^ in actual water samples using probe AH for spiked recovery experiment (*n* = 3).

Sample Type	Spiked (μM)	Detected (μM)	Recovery (%)	RSD (%)
Tap Water	0	Not detected	-	-
	4	4.28	107.00	3.09
	8	7.89	98.63	4.00
	12	12.34	102.83	3.61
River Water	0	Not detected	-	-
	4	3.72	93.00	2.84
	8	8.06	100.75	1.15
	12	12.38	103.17	2.84
Lake water	0	Not detected	-	-
	4	3.75	93.75	0.16
	8	8.36	104.50	3.87
	12	12.32	102.67	3.49

**Table 2:** Spike recovery tests for Cu^2+^ detection in real water samples using probe AH by the UV-vis method (*n* = 3).

Sample Type	Spiked (μM)	Detected (μM)	Recovery (%)	RSD (%)
Tap Water	0	Not detected	-	-
	4	4.13	103.25	0.57
	8	8.24	103.00	4.98
	12	12.16	101.33	0.29
River Water	0	Not detected	-	-
	4	3.97	99.25	1.31
	8	8.39	104.88	2.34
	12	11.96	99.67	0.31
Lake water	0	Not detected	-	-
	4	4.13	103.35	0.33
	8	8.47	105.88	1.45
	12	12.06	100.50	0.32

**Table 3:** A comparative analysis of reported Cu^2+^ probes with emphasis on probe AH.

Probe	Method	Solvent medium	λem/λmax	Response time	LOD	Reference
** 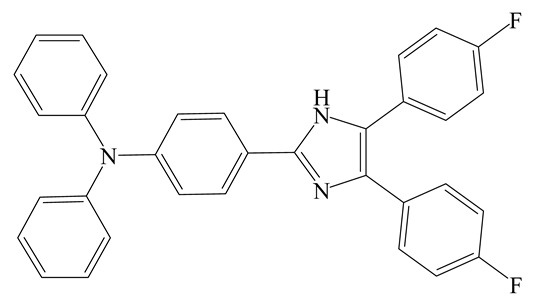 **	FL	MeCN	408 nm	4 min	3.93 µM	[2]
** 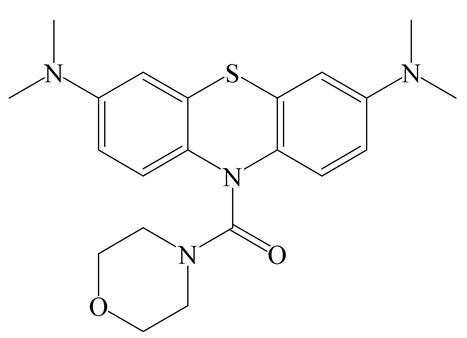 **	FL	MeCN/Tris-HCl (4:6)	689 nm	5 min	0.33 µM	[18]
** 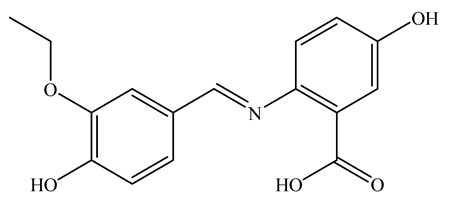 **	UV	EtOH/H_2_O (8:2)	275 nm (λmax)	Not mentioned	0.39 µM	[48]
** 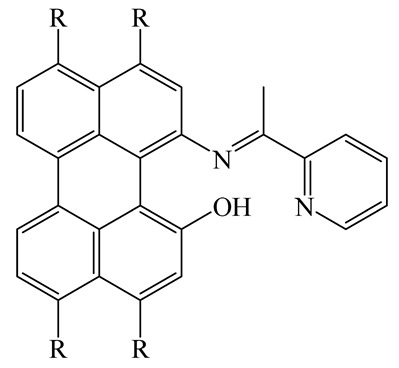 **	FL	DMSO	490 nm	3 min	0.89 µM	[49]
** 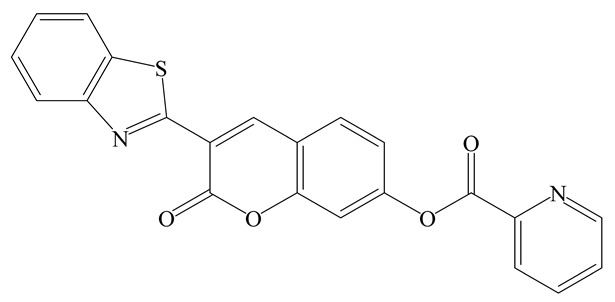 **	FL	DMSO/PBS (3:7)	493 nm	30 min	0.57 µM	[50]
** 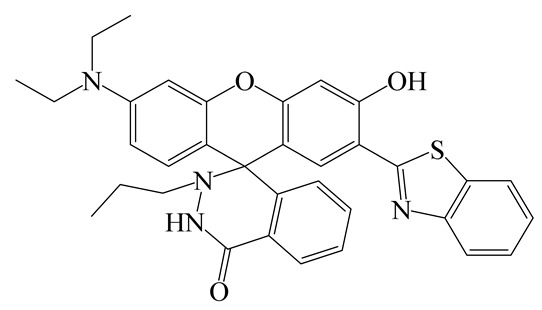 **	FL	MeCN/HEPES (3:7)	580 nm	40 min	0.25 µM	[51]
** 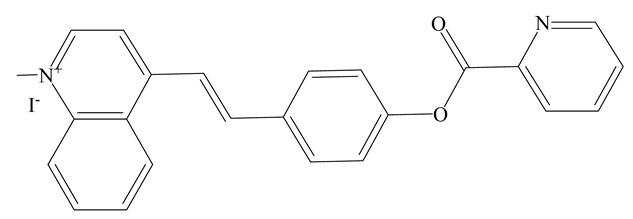 **	FL	HEPES	F560 nm/F495 nm	2 min	0.62 µM	[52]
** 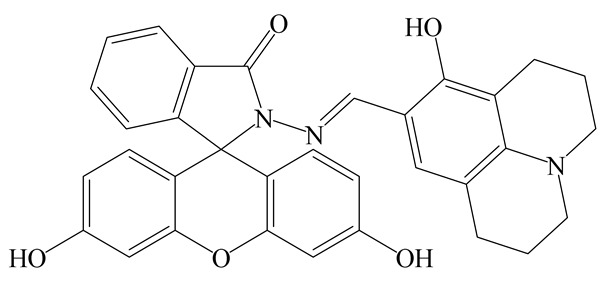 **	FL UV	EtOH/HEPES (9:1)	540 nm 452 nm (λmax)	1 min 1 min	0.22 µM 0.38 µM	This work

## Data Availability

Data will be provided by the authors upon request.

## Supplementary Materials

The following supporting information can be downloaded at: https://www.mdpi.com/article/10.3390/molecules30183824/s1, Figure S1: ^1^H-NMR (400 MHz, DMSO-*d*_6_) of compound **1**; Figure S2: ^13^C{^1^H} NMR (100 MHz, DMSO-*d*_6_) of compound **1**; Figure S3: IR spectrum of compound **1**; Figure S4: ^1^H-NMR (400 MHz, DMSO-*d*_6_) of probe AH; Figure S5: ^13^C{^1^H} NMR (100 MHz, DMSO-*d*_6_) of probe AH; Figure S6: IR spectrum of probe AH; Figure S7: HRMS spectrum of probe AH; Figure S8: Linear relationship between the A_452_/A_400_ ratio and Cu^2+^ concentration (0–12 μM; Figure S9: Assessment of the Cytotoxicity of AH (0–50 μM) on HepG2 cells; Figure S10: LSCM image of HepG2 cells incubated with AH (10 μM); Table S1: Spectral data of compounds AH and AH-Cu^2+^; Supporting Information of DFT Calculations; Table S2: The absolute electronic and corrected free energies of the optimized structures of intermediates and transition states for reaction calculated by the PCM B3LYP-D3/Def2-TZVP//B3LYP-D3/Def2-SVP method in ethanol solution.
