# Supplementary material for: A Novel Schiff Base Probe Based on Fluorescein for Fluorometric and Colorimetric Dual-Mode Rapid Detection of Cu2+
Source: Molecules. 2025 Sep 20;30(18):3824. doi: 10.3390/molecules30183824 (PMC12472230; doi:10.3390/molecules30183824)
Supplement: Supplementary file 1 [file molecules-30-03824-s001.zip › molecules-3695462-supplementary.pdf]

Supplementary Information

A Novel Schiff Base Probe Based on Fluorescein for Fluorometric and Colorimetric Dual-Mode Rapid Detection of Cu^{2+}

Zhi Yang [1,2], Chaojie Lei [2], Qian Wang [1,*], Yonghui He [2] and Senlin Tian [1,*]

[1] Faculty of Environmental Science and Engineering, Kunming University of Science and Technology, Kunming 650500, China; ymu777@163.com (Z. Y.)
[2] National and Local Joint Engineering Research Center for Green Preparation Technology of Biobased Materials, Yunnan Minzu University, Kunming 650500, China; xatutougao@163.com (C.L.); heyonghui@ymu.edu.cn (Y.H.)
* Correspondence: drifting_leaf@126.com (Q.W.); tiansenlin@outlook.com (S.T.); Tel.: +86-18706700561 (Q.W.)

Supporting figure captions/legends:

Figure S1: ^{1}H-NMR (400 MHz, DMSO-d_6) of compound **1**.

Figure S2: ^{13}C{^{1}H} NMR (100 MHz, DMSO-d_6) of compound **1**.

Figure S3: IR spectrum of compound **1**.

Figure S4: ^{1}H-NMR (400 MHz, DMSO-d_6) of probe AH.

Figure S5: ^{13}C{^{1}H} NMR (100 MHz, DMSO-d_6) of probe AH.

Figure S6: IR spectrum of probe AH.

Figure S7: HRMS spectrum of probe AH.

Table S1: Spectral data of compounds AH and AH-Cu^{2+}.

Supporting Information of DFT Calculations.

Table S2: The absolute electronic and corrected free energies of the optimized structures of intermediates and transition states for reaction calculated by the PCM B3LYP-D3/Def2-TZVP//B3LYP-D3/Def2-SVP method in ethanol solution.

Figure S8: Linear relationship between the A_{452}/A_{400} ratio and Cu^{2+} concentration (0−12 μM).

Figure S9: Assessment of the Cytotoxicity of AH (0–50 μM) on HepG2 cells.

Figure S10: LSCM image of HepG2 cells incubated with AH (10 μM).

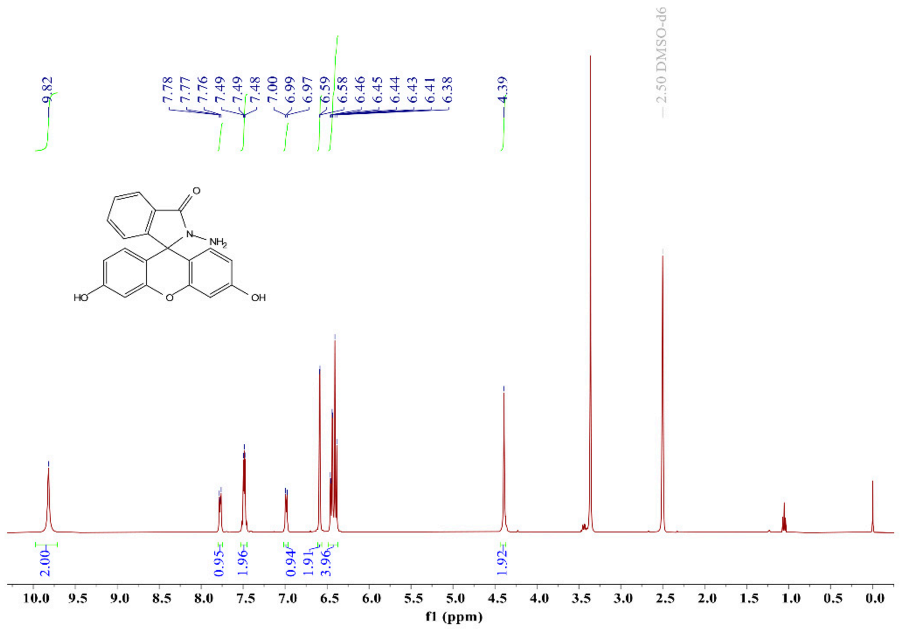

Figure S1. ^{1}H-NMR (400 MHz, DMSO-*d6*) of compound 1.

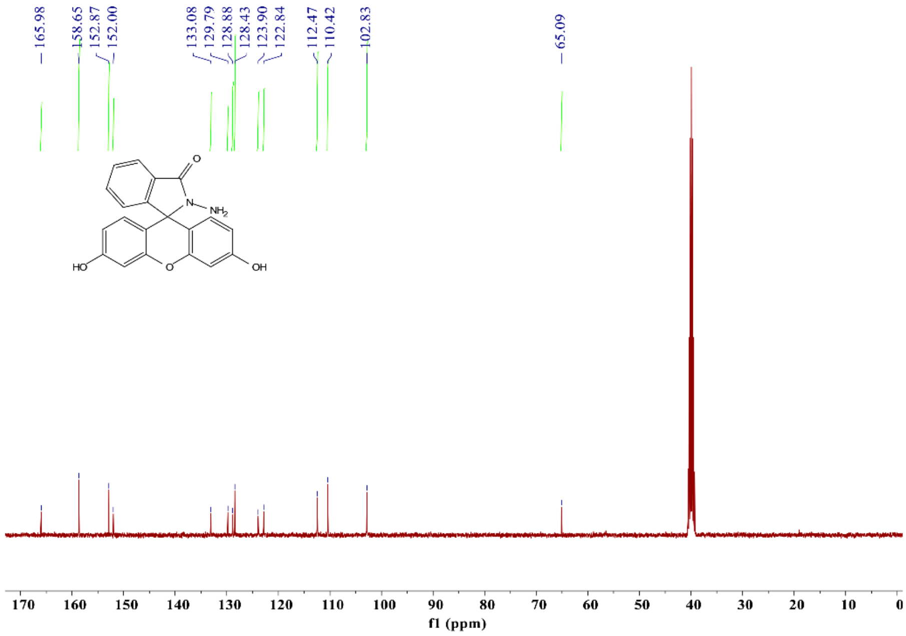

Figure S2. $^{13}C\{^{1}H\}$ NMR (100 MHz, DMSO-*d6*) of compound 1.

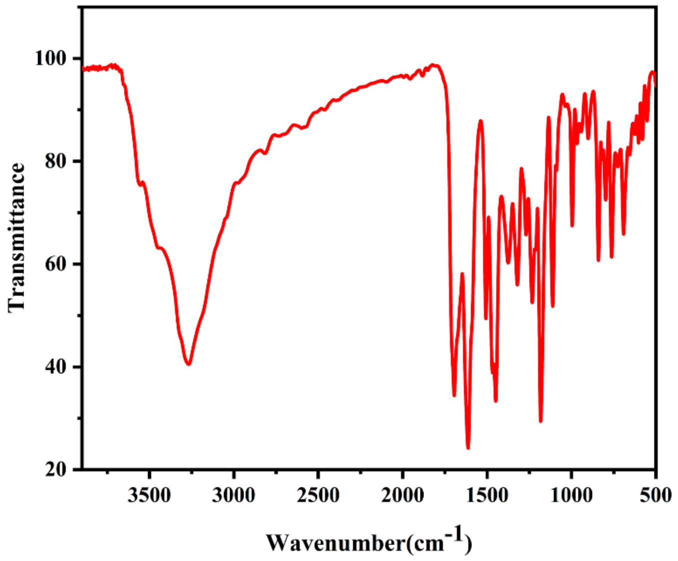

Figure S3. IR spectrum of compound 1.

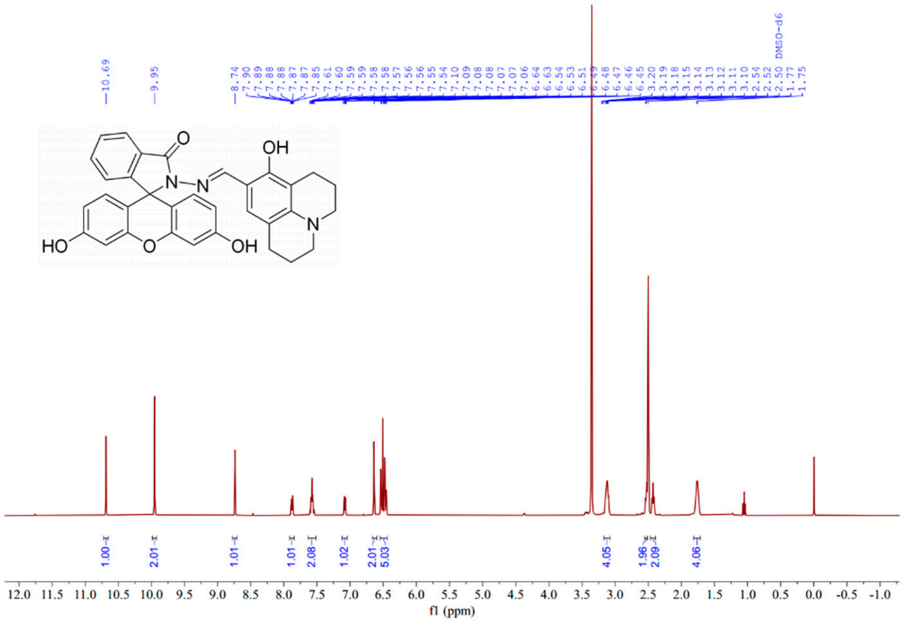

Figure S4. ^{1}H-NMR (400 MHz, DMSO-*d6*) of probe AH.

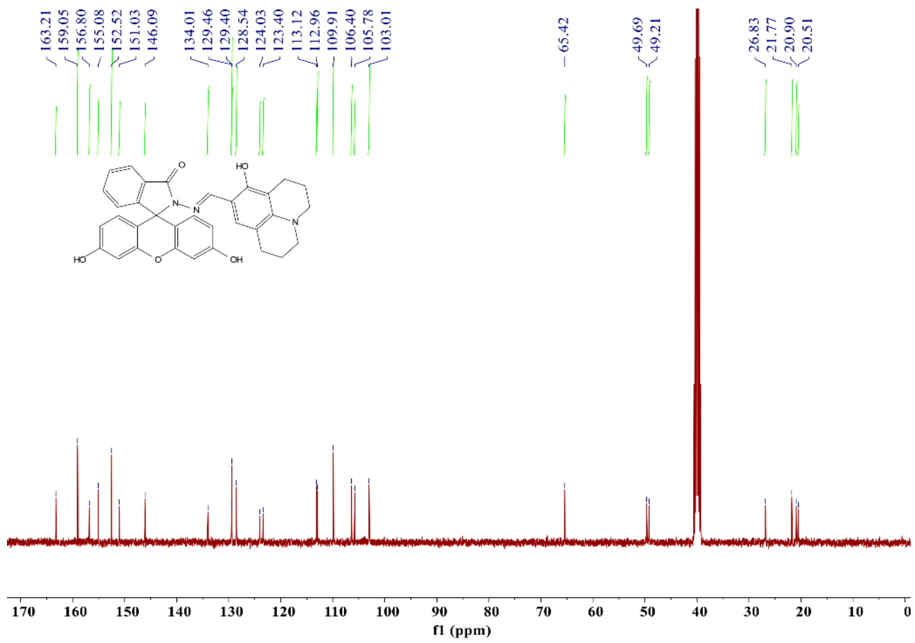

Figure S5. $^{13}C\{^{1}H\}$ NMR (100 MHz, DMSO-*d6*) of probe AH.

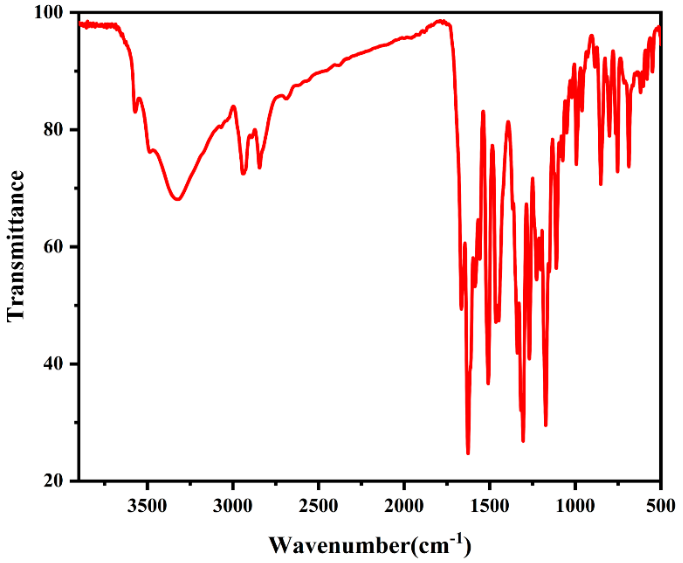

Figure S6. IR spectrum of probe AH.

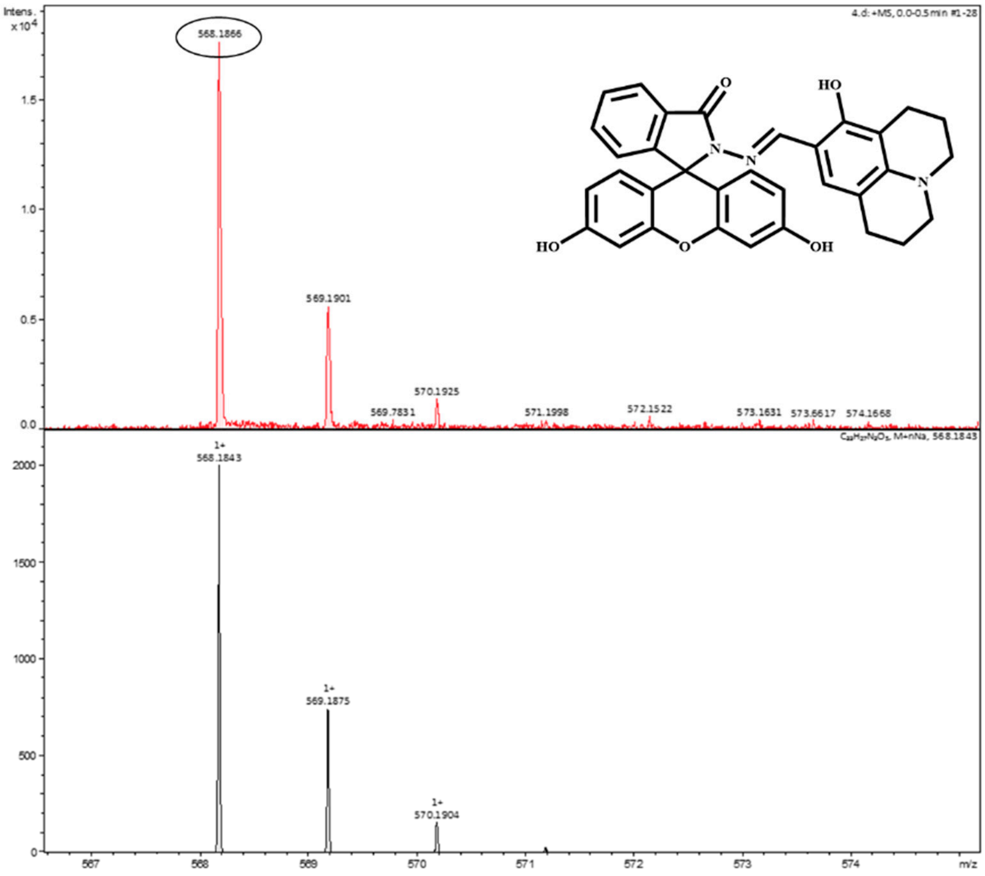

Figure S7. HRMS spectrum of probe AH.

Table S1. Spectral data of compounds AH and AH-Cu^{2+}.

**Compounds**	**λ max (nm)**	**ε (L • moL^{-1} • cm^{-1})**	**λ ex (nm)**	**λ em (nm)**	**Stokes shift (nm)**	**Φ**
AH	400	3.42×10^4	400	536	136	0.133
AH-Cu^{2+}	452	3.84×10^4	400	540	138	0.001

Supporting Information of DFT Calculations

Computational methods

All density functional theory (DFT) calculations were performed using B3LYP-D3[1] method combined with a Def2-SVP basis set[2] for all atoms were used to fully optimize all structures in the gas-phase. Then, vibrational frequency calculations on these optimized geometries were carried out at the same level of theory to confirm no imaginary frequency for all local minimum, and one appropriate imaginary frequency for each transition state. To obtain higher accurate energies, the Def2-TZVP basis set was also used for the single-point energy calculations in PCM solvent model[3] using ethanol solution. All DFT calculations were carried out by Gaussian16 program.[4] All 3D images of the optimized structures were shown by CYLview.[5] All free energies are

corrected with Grimme's quasi-harmonic approximation for vibrational entropy correction at all frequencies below 100 cm^{-1} by GoodVibes using default settings.[6]

References

[1] (a) Lee, C.; Yang, W.; Parr, R. G. Development of the Colle-Salvetti correlation-energy formula into a functional of the electron density. Phys. Rev. B. 1988, 37, 785-789; (b) Becke, A. D. Density-functional thermochemistry. III. The role of exact exchange. J. Chem. Phys. 1993, 98, 5648-5652; (c) Grimme, S.; Antony, J.; Ehrlich, S.; Krieg, H. A consistent and accurate ab initio parametrization of density functional dispersion correction (DFT-D) for the 94 elements H-Pu. J. Chem. Phys. 2010, 132, 154104.

[2] Weigend, F.; and Ahlrichs, R. Balanced basis sets of split valence, triple zeta valence and quadruple zeta valence quality for H to Rn: Design and assessment of accuracy. Phys. Chem. Chem. Phys. 2005, 7, 3297–3305.

[3] (a) Tomasi, J.; Mennucci, B.; Cammi, R. Quantum Mechanical Continuum Solvation Models. Chem. Rev. 2005, 105, 2999-3094; (b) Scalmani, G.; Frisch, M. J. Continuous surface charge polarizable continuum models of solvation. I. General formalism. J. Chem. Phys. 2010, 132, 114110.

[4] Gaussian 16, Revision A.03, Frisch, M. J.; Trucks, G. W.; Schlegel, H. B.; Scuseria, G. E.; Robb, M. A.; Cheeseman, J. R.; Scalmani, G.; Barone, V.; Mennucci, B.; Petersson, G. A.; Nakatsuji, H.; Caricato, M.; Li, X.; Hratchian, H. P.; Izmaylov, A. F.; Bloino, J.; Zheng, G.; Sonnenberg, J. L.; Hada, M.; Ehara, M.; Toyota, K.; Fukuda, R.; Hasegawa, J.; Ishida, M.; Nakajima, T.; Honda, Y.; Kitao, O.; Nakai, H.; Vreven, T.; Montgomery, J. A., Jr.; Peralta, J. E.; Ogliaro, F.; Bearpark, M.; Heyd, J. J.; Brothers, E.; Kudin, K. N.; Staroverov, V. N.; Kobayashi, R.; Normand, J.; Raghavachari, K.; Rendell, A.; Burant, J. C.; Iyengar, S. S.; Tomasi, J.; Cossi, M.; Rega, N.; Millam, J. M.; Klene, M.; Knox, J. E.; Cross, J. B.; Bakken, V.; Adamo, C.; Jaramillo, J.; Gomperts, R.; Stratmann, R. E.; Yazyev, O.; Austin, A. J.; Cammi, R.; Pomelli, C.; Ochterski, J. W.; Martin, R. L.; Morokuma, K.; Zakrzewski, V. G.; Voth, G. A.; Salvador, P.; Dannenberg, J. J.; Dapprich, S.; Daniels, A. D.; Farkas, Ö.; Foresman, J. B.; Ortiz, J. V.; Cioslowski, J.; Fox, D. J. Gaussian, Inc., Wallingford CT, 2016.

[5] Legault, C. Y. CYL View, version 20; Universite de Sherbrooke, Sherbrooke, Quebec, Canada, 2020; https://www.cylview.org.

[6] Luchini, G.; Alegre-Requena, J. V.; Funes-Ardoiz I.; Paton, R. S. GoodVibes: automated thermochemistry for heterogeneous computational chemistry data. F1000Research. 2020, 9, 291.

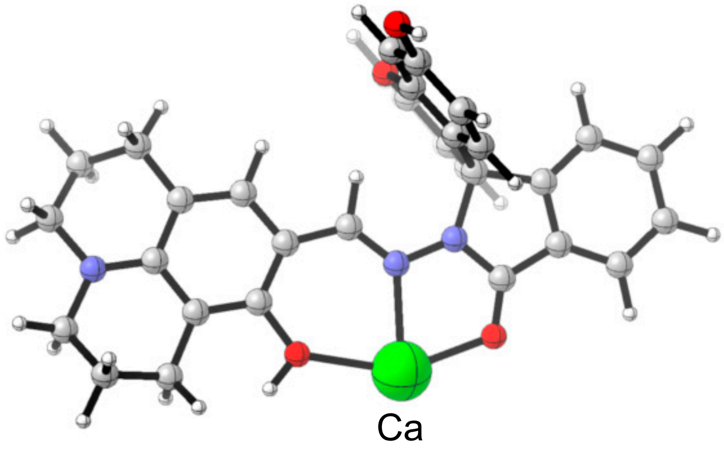

Binding Energy: −3.3 kcal/mol

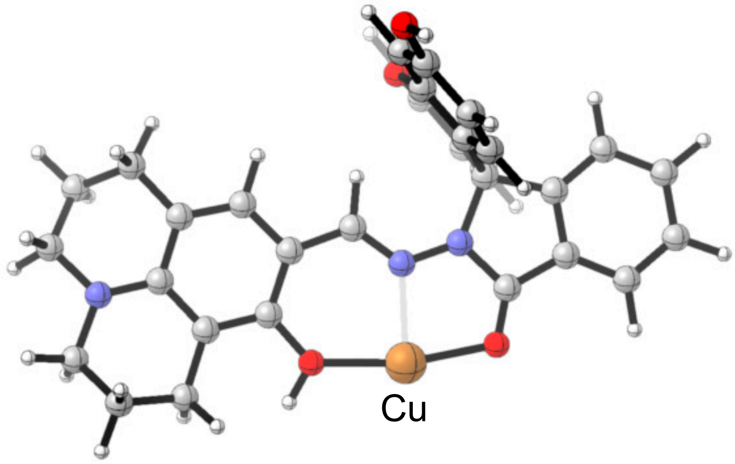

Binding Energy: −145.6 kcal/mol

Table S2. The absolute electronic and corrected free energies of the optimized structures of intermediates and transition states for reaction calculated by the PCM B3LYP-D3/Def2-TZVP//B3LYP-D3/Def2-SVP method in ethanol solution. (The free energies are corrected with quasi-harmonic entropy correction.)

	E_{gas} (Hartree)	E_{soln} (Hartree)	G_{gas}-qh (Hartree)	DG_{soln}-qh (kcal/mol)
**AH**	-1812.94670	-1814.93711	-1812.46380	
**Ca^{2+}**	-676.81984	-677.45013	-676.83505	
**AH- Ca^{2+}**	-2490.10224	-2492.40947	-2489.61762	−3.3
**Cu^{2+}**	-1639.24826	-1640.01090	-1639.26477	
**AH- Cu^{2+}**	-3452.79985	-3455.19638	-3452.31709	−145.6

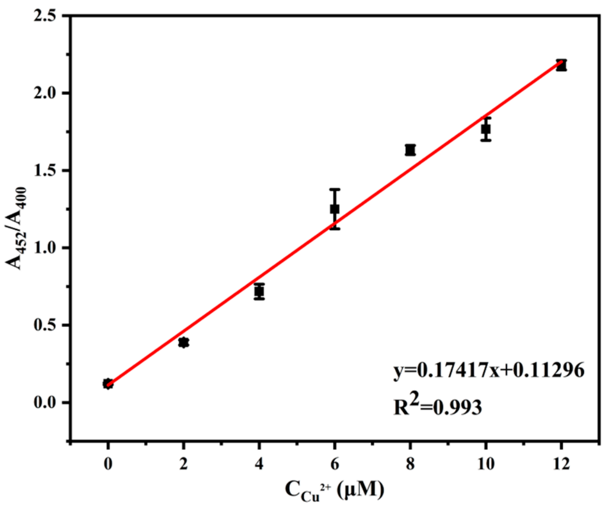

Figure S8. Linear relationship between the A_{452}/A_{400} ratio and Cu^{2+} concentration (0−12 μM).

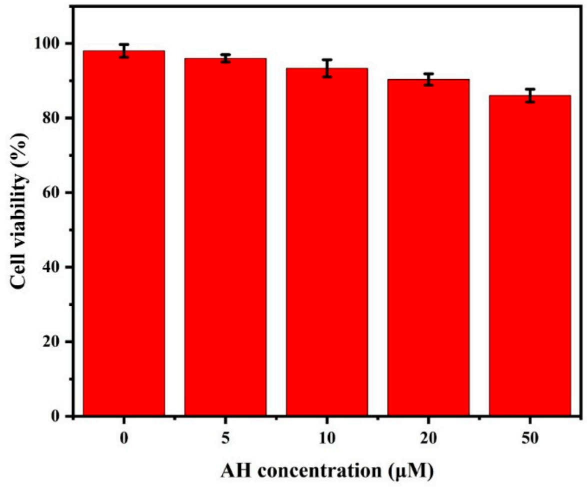

Figure S9. Assessment of the Cytotoxicity of AH (0–50 μM) on HepG2 cells.

With regard to Figure S9, the cytotoxicity of probe AH was evaluated using the MTT assay. HepG2 cells were seeded in 96-well plates and exposed to varying concentrations of AH (0–50 μM). Following incubation at 37 °C for 24 hours, MTT solution was added, and the cells were further incubated for 4 hours. Subsequently, the supernatant was removed, and DMSO was added to dissolve the formed formazan crystals. Finally, the absorbance was measured using a microplate reader at a wavelength of 490 nm, and the cell viability (%) was calculated based on the absorbance values.

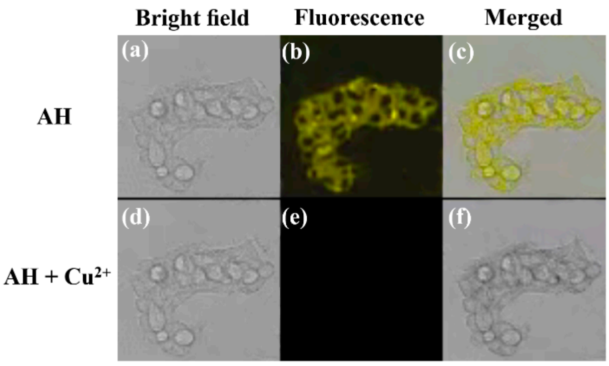

Figure S10. LSCM image of HepG2 cells incubated with AH (10 μM).

With regard to Figure S10, HepG2 cells were cultured in DMEM medium supplemented with 10% fetal bovine serum, 100 units/mL penicillin, and 100 mg/L streptomycin, and incubated at 37°C in a humidified atmosphere containing 5% CO_2 for 24 hours. Subsequently, probe AH (10 μM) or Cu^{2+} (12 μM) solution was added, and the cells were further incubated for 2 hours. Finally, cellular imaging was performed using a laser scanning confocal microscope.
